# Attention to sound improves auditory reliability in audio-tactile spatial optimal integration

**DOI:** 10.3389/fnint.2015.00034

**Published:** 2015-05-07

**Authors:** Tiziana Vercillo, Monica Gori

**Affiliations:** Robotics, Brain, and Cognitive Sciences Department, Fondazione Istituto Italiano di TecnologiaGenoa, Italy

**Keywords:** attention, multisensory integration, auditory, bayes theorem, sensory cue

## Abstract

The role of attention on multisensory processing is still poorly understood. In particular, it is unclear whether directing attention toward a sensory cue dynamically reweights cue reliability during integration of multiple sensory signals. In this study, we investigated the impact of attention in combining audio-tactile signals in an optimal fashion. We used the Maximum Likelihood Estimation (MLE) model to predict audio-tactile spatial localization on the body surface. We developed a new audio-tactile device composed by several small units, each one consisting of a speaker and a tactile vibrator independently controllable by external software. We tested participants in an attentional and a non-attentional condition. In the attentional experiment, participants performed a dual task paradigm: they were required to evaluate the duration of a sound while performing an audio-tactile spatial task. Three unisensory or multisensory stimuli, conflictual or not conflictual sounds and vibrations arranged along the horizontal axis, were presented sequentially. In the primary task participants had to evaluate in a space bisection task the position of the second stimulus (the probe) with respect to the others (the standards). In the secondary task they had to report occasionally changes in duration of the second auditory stimulus. In the non-attentional task participants had only to perform the primary task (space bisection). Our results showed an enhanced auditory precision (and auditory weights) in the auditory attentional condition with respect to the control non-attentional condition. The results of this study support the idea that modality-specific attention modulates multisensory integration.

## Introduction

Spatio-temporal coincident sensory signals are combined together to generate multisensory percepts. Sensory information is weighted accordingly to its reliability and integrated in a statistically optimal fashion (Clarke and Yuille, 1990; Ghahramani et al., 1997; Ernst and Banks, 2002; Alais and Burr, 2004; Landy et al., 2011). Although years of intensive studies have produced a wide body of research on the topic of multisensory integration, it is still unclear whether or not attended stimuli are integrated differently from those that are not attended. Specifically, it is not clear whether the mechanism of multisensory integration occurs automatically and pre-attentively or whether attention affects the sensory binding. Several studies support the first idea, reporting differences in the perceptual estimates when people attend to one or another sensory modality in a multisensory task (Bertelson and Radeau, 1981; Warren et al., 1981). For example, Oruc et al. (2008) demonstrated that crossmodal dynamic ventriloquism (Soto-Faraco et al., 2002), the illusory reversal in the perceived direction of motion of a target modality induced by the opposite motion direction of a distractor modality, can be affected by modality-specific attention. Similarly in another study, Alsius et al. (2005) reported that the audio-visual McGurk illusion is powerfully reduced when participants perform a concurrent auditory or visual task, suggesting that the high attentional load precludes multisensory processing.

Differently, other studies found that attention has no effect on multisensory integration, supporting the idea that sensory cues are combined pre-attentively. For example, Driver (1996) showed that the ventriloquist cross-modal illusion can enhance selective spatial attention to speech sounds, suggesting that the multisensory binding has to occur before the auditory attentive selection. Furthermore, other studies suggest that there are no effects of endogenous (Bertelson et al., 2000) and automatic visual attention (Vroomen et al., 2001) on audio-visual ventriloquism. Bertelson et al. (2000) reported no effect of attention when participants had to localize the apparent source of a sound presented with a synchronous peripheral flash while monitoring occasional slight changes in shape of a visual target in a central or in a peripheral position, supporting the idea that multisensory integration is a pre-attentive process (Driver, 1996; Vroomen et al., 2001).

Although a great deal of consideration has been paid to the effect of attention on multisensory processing, there is much less effort directed to quantify such effects with the Maximum Likelihood Estimation (MLE) model. Helbig and Ernst (2008) have recently investigated the effects of modality-specific attention on multisensory optimal integration, adopting a dual task paradigm. Participants were asked to evaluate similarities or differences between two sequences of letters while performing a visual-haptic size discrimination task. Participants' performance was later compared to an ideal observer (MLE model) to test for optimal integration. Results showed no effect of modality-specific attention on visual-haptic optimal integration, sustaining the hypothesis that the mechanism of integration is pre-attentive. Visual and tactile weights were untouched by the distractor task. Furthermore, the bimodal JNDs, although increased in the dual task condition, were still lower than both of the unisensory JNDs, as predicted by the MLE model.

Interestingly, the distractor task used by Helbig and Ernst (2008), and by several other studies to date (Alsius et al., 2005) involved the use of stimuli with qualitatively different properties from those used in the primary task. A possible reason for the absence of attentional effects on multisensory integration could be that the simultaneous encoding of qualitatively different stimuli (e.g., size vs. letter) increases the attentional load, rather than focusing attention on a sensory modality.

Here we examined the attentional modulation of multisensory integration in a dual task where the same stimulus had to be evaluated twice. Participants were asked to execute an acoustic temporal discrimination task while performing an audio-tactile spatial bisection task. Recent researches reported that audition and touch can interact pre-attentively. Butler et al. (2012) demonstrated audio-tactile pre-attentive interaction at the cortical level during frequency processing. Yau et al. (2009) reported that auditory stimuli can interfere with tactile frequency perception when auditory and tactile stimuli share similar frequencies. Of greater interest for our study is that audio-tactile integration seems to vary according to the perceptual task that participants have to perform. Yau et al. (2010) reported separate integration mechanisms for audio-tactile interactions in frequency and intensity perception. While the effects of sensory capture appear to be stronger and pre-attentive for frequency perception, suggesting shared processing for spectral analysis, audio-tactile interactions for intensity discrimination depend on the attended modality.

We investigated the effect of attention on auditory precision and multisensory optimal processing when participants had to simultaneously evaluate an auditory stimulus in two different domains (temporal and spatial) while integrating it with a tactile signal in the spatial (and not in the temporal) domain. We expected that the simultaneous estimation of multiple characteristics of the same stimulus may affect its reliability during multisensory integration. Moreover we compared the performance of all the participants with an optimal estimator.

## Methods and procedures

### Participants

Ten adults (28 ± 1 years of age) participated at experiment. All of them had normal hearing. Participants were blindfolded before entering the room, so they had no notion of the experimental setup. All participants signed informed consents before starting the experiment. Testing procedures were approved by the ASL3 of Genoa (Italy).

### Stimuli

**Figure 1:**
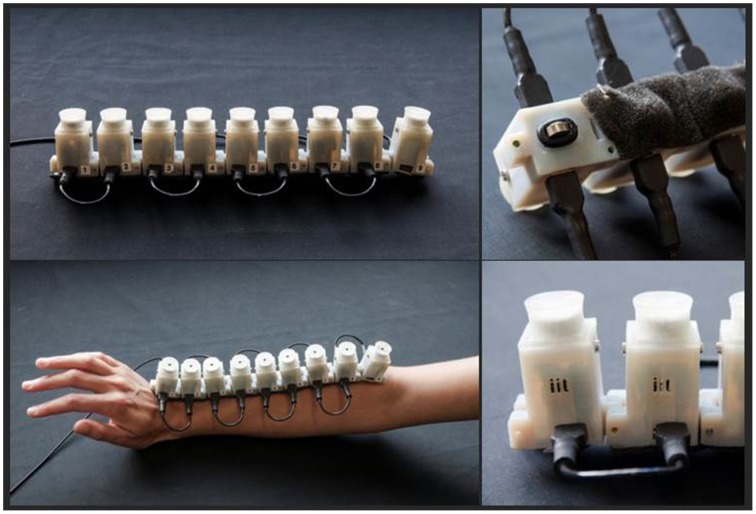
**Images from the device used during the test**. It is composed by 9 units constituted by a speaker and a vibrating motor individually controllable. Units were separated by 3.5 cm. The device was located on the forearm, with the the 1st unit close to the hand and the 9th unit close to elbow.

For the audio-tactile stimulation we developed a device composed by 9 units which could be controlled individually. Units were separated by 3.5 cm (11° of visual angle). Each unit was composed by a speaker producing a 2978 Hz pure tone associated to a 2V vibrating motor (Figure 1). The vibrotactile motors produced tactile stimulation of 120 Hz, with vibration amplitude of 0.55 G.

### Procedures

Participants placed their right arm on a support at the eyes' level, at a distance of 18 cm from their eyes. The device was positioned on the forearm, with the 5th unit (the middle of the array) aligned with the nose, the 1st unit close to the hand (the left side of participants' head) and the 9th unit close to elbow (at the right side of participants' head). Participants wore acoustic earmuffs (Howard leight, Viking™ V1) during all the experiment, to attenuate the noise emitted by the vibrotactile stimulator while hearing sounds at ordinary volumes and frequencies normally.

Two tests were performed. In the non-attentional condition we measured discrimination thresholds and PSEs in a spatial bisection task. Only-audio, only-tactile and audio-tactile stimuli were provided. For each trial, we presented a sequence of three stimuli (auditory, tactile or both) for a total duration of 1.7 s, with the second and the third stimuli occurring always 600 ms after the onset of the previous stimulus. The duration of the auditory and the tactile stimuli was 500 ms. The locations of the first and the third stimulus (standard stimuli) of the sequence were fixed at the 1st (−14 cm) and the 9th (+14 cm) units, respectively, while the location of the second stimulus was controlled by the adaptive QUEST algorithm (Watson and Pelli, 1983). The QUEST algorithm estimates PSE after each response and places the next trial near that estimate. To ensure that a wide range of positions was sampled, that estimate was jittered by a random amount, drawn from a Gaussian distribution of space constant 10 cm, and the nearest unit to that estimate chosen. We will refer to the position estimated by the QUEST algorithm as the “probe.”

In the unisensory tasks participants were presented with a sequence of three vibrotactile stimulations or sounds and participants had to report whether the second stimulus appeared closer in space to the first or the third stimulus. The second auditory or tactile stimulus was placed at the position estimated by the QUEST algorithm (the probe). In the bimodal task, the sequence of three vibrotactile signals was associated to three sounds. In this last condition, the second stimulus could have been presented in conflict with auditory and tactile stimuli located in different positions and at different distances from the probe. The audio-tactile conflict (Δ) was calculated as S_A_–S_T_, with S_A_and S_T_ representing the spatial distance of the auditory and the tactile stimuli with respect to the probe (see Alais and Burr, 2004; Gori et al., 2012). In the no-conflict condition (Δ = 0 cm), the location of the auditory and the tactile stimulus corresponded to the probe. In the conflict conditions (Δ = ±7 cm), auditory and tactile stimuli were presented at ±3.5 cm from the probe. For example if the probe was 0 (the fifth unit, the center of the device), in the Δ = +7 cm condition the sound was located at +3.5 cm and the vibration at −3.5 cm [3.5 − (−3.5) = 7 cm]; conversely, in the Δ = −7 condition the sound was located at −3.5 cm while the vibration at +3.5 cm [−3.5 − (3.5) = −7]. In the case that the probe was estimated in a position outside of the stimulus array, the closest unit to the extreme position was selected. Therefore, the second auditory or tactile stimuli could have been presented also in the two extremes locations. In the first and the third audio-tactile stimulus, the auditory and tactile components were presented aligned, with no spatial conflict.

Participants performed 90 trials for both the unisensory conditions and 90 trials for each conflict in the bimodal condition. Conditions were mixed within each block, and presented in a random order. Data for each condition were fitted with cumulative Gaussians. The proportion of rightward responses was plotted as a function of the speaker position, and the data fitted with a Cumulative Gaussian function by means of the Maximum Likelihood method to estimate both PSE (point of subjective equality, given by the mean) and threshold (standard deviation). The space constant (σ) of the fit was taken as the estimate of threshold indicating precision for the bisection task. Standard errors in the threshold and PSEs were computed with bootstrap simulation (Efron and Tibshirani, 1993). All conflict conditions were used to obtain the bimodal threshold estimates. Despite the audio-tactile conflict the stimulation appeared as a single stimulus; participants did not notice the conflict even when asked. Unimodal and bimodal (conflictual or not) audio-tactile thresholds and PSEs were compared with the prediction of the MLE model.

In the attentional condition we introduced an auditory dual task to focus participants' attention only in the auditory stream. This time in addition to the spatial bisection task, participants were also asked to identify occasionally changes in duration of the second auditory stimulus. The duration of the second sound was manipulated only in the 30% of the trials (catch trials) for each block. The task was extremely easy to perform since the second sound might have been 150 ms longer or shorter than its normal duration and than the other two sounds of the sequence. All these catch trials were excluded from the data analysis. The remaining data for each condition were fitted with cumulative Gaussians. Unimodal and bimodal audio-tactile thresholds and PSEs were compared with the prediction of the MLE model. Participants performed the same amount of trials as they did in the non-attentional condition. The order of the two attentional conditions was counterbalanced across participants.

## Maximum likelihood model

The MLE calculation assumes that the optimal bimodal estimate of PSE ($\hat{S}$_*AT*_) is given by the weighted sum of the independent audio and tactile estimates ($\hat{S}$_*A*_ and $\hat{S}$_*T*_).

(1) $${\hat{S}}_{AT}=w_{A}{\hat{S}}_{A}+w_{T}{\hat{S}}_{T}$$

Where weights *w_A_* and *w_T_* sum to unity and are inversely proportional to the variance (σ^2^) of the underlying noise distribution, assessed from the standard deviation σ of the Gaussian fit of the psychometric functions for audio and tactile judgments:

(2) $$w_{A}=\sigma_{T}^{2}/\left(\sigma_{T}^{2}+\sigma_{A}^{2}\right),w_{T}=\sigma_{A}^{2}/\left(\sigma_{T}^{2}+\sigma_{A}^{2}\right)$$

The MLE prediction for the audio-tactile threshold (σ_*AT*_) is given by:

(3) $$\sigma_{AT}^{2}=\frac{\sigma_{A}^{2}\sigma_{T}^{2}}{\sigma_{A}^{2}+\sigma_{T}^{2}}\leq \min\left(\sigma_{A}^{2},\sigma_{T}^{2}\right)$$

where σ_*A*_ and σ_*T*_ are the audio and tactile unimodal thresholds. The improvement is greatest ($\sqrt{2}$) when σ_*A*_ = σ_*T*_.

To calculate the audio and tactile weights from the PSEs, we substituted the actual second sound position (relative to standard) into Equation (1):

(4) $$\hat{S}\left(\Delta \right)=\left(w_{A}\Delta -w_{T}\Delta \right)=\left(1-2w_{T}\right)\Delta$$

The slope of the function is given by the first derivative:

(5) $$\hat{S}(\Delta {)}^{\prime }=1-2w_{T}$$

Rearranging:

(6) $$w_{T}=\left(1-\hat{S}(\Delta {)}^{\prime }\right)/2$$

The slope $\hat{S}$(Δ)′ was calculated by linear regression of PSEs for all values of Δ, separately for each subject and each condition.

## Results

### Unisensory tasks

**Figure 2:**
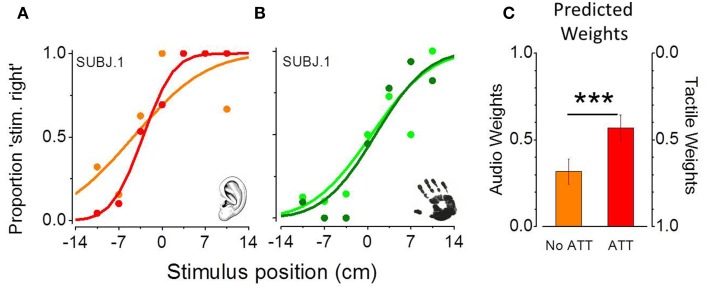
**Psychometric functions from one representative subject in the only-audio (A) and the only-tactile condition (B)**. The curves plot proportion of trials where the second stimulus was perceived more on the right for all its positions. Light green and light red curves represent subject's performance for the non-attentional condition and dark green and dark red curves the performance for the attentional condition. Data were fitted with cumulative Gaussians, to obtain PSE (50%) and threshold (standard deviation of the best-fitting function). Thresholds decrease in the attentional task only for the auditory condition. **(C)** Shows predicted average audio and tactile weights, calculated from individual thresholds in the two experimental conditions. Auditory weights are higher in the attentional condition. (^***^p < 0.001).

Figures 2A,B show psychometric functions from one representative subject in the only-audio and in the only-tactile condition. Each function describes the proportion of trials where the second stimulus was perceived more on the right for all its spatial locations. Light green and light red curves represent the performance of the subject in the non-attentional task and dark green and dark red curves the performance in the attentional task. The point of subjective equality (PSE) represents the stimulus position that participants judged as more on the right in 50% of the trials. The slopes of the psychometric functions, given by the standard deviations of the best-fitting Gaussian error function, provide an estimate of the precision in the spatial task. The steeper the curve, the higher the precision. We mainly based the statistical analysis on these two measures, as described below. Looking at the thresholds (the slopes of the psychometric functions in Figures 2A,B, that are also reported in **Figures 4C,D**) it is clear that in the non-attentional condition, participants performed the tactile task with higher precision than the auditory one [one tailed paired *t*-test; *t*_(9)_ = 2.08; *P* = 0.03]. Interestingly, we found that the temporal auditory task improves auditory precision in the spatial task [one tailed paired *t*-test; *t*_(9)_ = 1.88; *P* = 0.04] and declines the tactile precision [one tailed paired *t*-test;*t*_(9)_ = −2.17; *P* = 0.02]. The improved auditory precision, and the lack of significant difference between auditory and tactile thresholds in the attentional condition [one tailed paired *t*-test; *t*_(9)_ = 1.18; *P* = 0.86] result in a large enhancement of the predicted auditory weights in the attentional condition with respect to the non-attentional condition [one tailed paired *t*-test; *t*_(9)_ = 5.55; *P* = 0.001]. Figure 2C shows predicted average audio and tactile weights, calculated from all the individual thresholds in the two experimental conditions. Predicted auditory weights in the non-attentional condition were equal to 0.32 ± 0.07 and become equal to 0.57 ± 0.07 in the attentional condition. Interestingly, predicted tactile weights vary from 0.68 ± 0.07, in the non-attentional condition, to 0.43 ± 0.21 in the attentional condition. Following the MLE model (Equation 1) we should expect tactile dominance in the non-attentional condition and auditory dominance or no dominance in the attentional condition.

### Bimodal tasks

**Figure 3:**
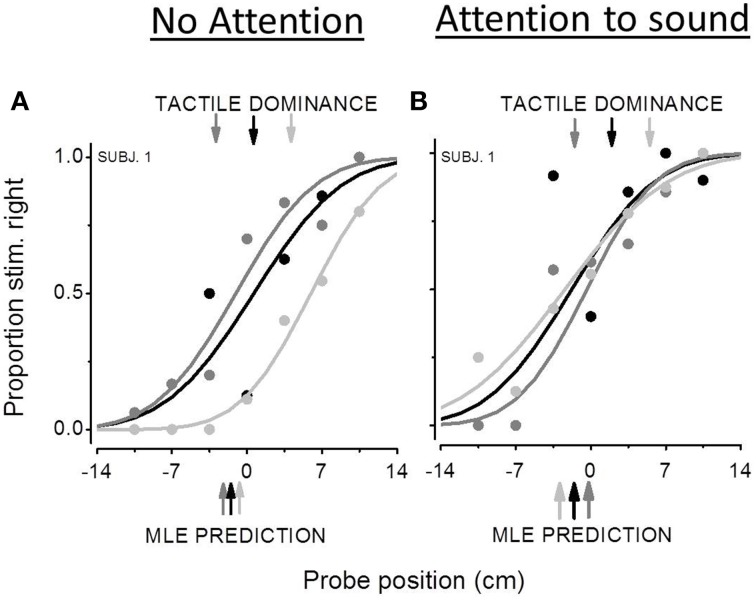
**Bimodal psychometric functions from the same participant in the non attentional condition **(A)** and in the attentional condition **(B)****. The black curve represents subject's performance in the Δ = 0 audio-tactile conflict, the dark gray curve the performance in the Δ = −7 conflict and the light gray curve the Δ = +7 conflict. The x-axis represents the estimated position of the second stimulus by the QUEST algorithm (cm). Positive values of PSE mean a spatial bias to the left, while negative values a bias to the right. The lower color-coded arrows show the MLE prediction. The upper color-coded arrows indicate the predicted PSEs in the case of tactile dominance.

Figure 3 reports bimodal psychometric functions from the same representative subject for the three audio-tactile conflicts: Δ = 0, Δ = −7 (dark gray curve), Δ = +7 (light gray curve). The proportion of the “stimulus more on the right” responses is plotted as a function of the estimated position of the probe (the location calculated by the QUEST algorithm). Positive values of the PSE mean that participants are following the modality presented at −3.5 cm from the probe. For example in the conflict condition of Δ = +7, a positive PSE means that subject are founding their perceptual estimates on the tactile modality. Indeed when the probe is higher than 0, the auditory stimulus is located at +3.5 cm, then closer to the extreme right, while the tactile stimulus is closer to the 0 position. Conversely, negative values of the PSE mean that participants are founding their perceptual estimates on the sensory modality presented at +3.5 cm with respect to the probe. The lower color-coded arrows show the MLE prediction. The upper color-coded arrows indicate the predicted PSEs in the case of tactile dominance. Results from the first experiment (Figure 3A) showed poor audio-tactile integration with tactile dominance. In the Δ = −7 condition, when the auditory stimulus is located more on the left than the tactile one, the psychometric curve is shifted toward negative value, denoting a bias to the right in the direction of the tactile stimulus. In this condition, as well as the Δ = 0 condition, measured PSEs are very similar to PSEs predicted by the MLE model. Conversely, in the Δ = +7 condition, the psychometric function is shifted toward positive values implying a bias to the left in the direction of the tactile stimulus and the measured PSE is closer to the one predicted in the case of tactile dominance. In the auditory attentional condition, the bimodal psychometric functions are in the inverted position; however, they are all fairly centered on the 0 confirming that the two sensory modalities share similar weights.

**Figure 4:**
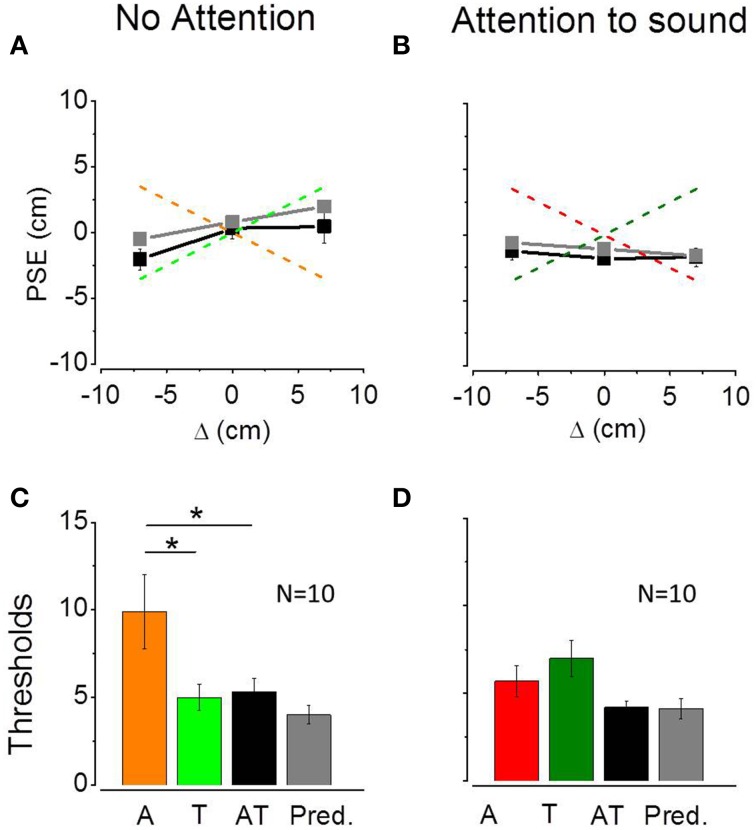
**(A,B)** Average PSEs (*N* = 10) for the three bimodal conflicts plotted as a function of the audio-tactile conflict (Δ) in the non-attentional condition **(A)** and in the attentional condition **(B)**. Error bars represent standard errors of the mean. Red and green lines describe the ideal performance in the case of auditory or tactile dominance, respectively. Black line and symbols show measured data, while gray line and symbols describe the model prediction. (**C,D**) Average thresholds for the audio (A), tactile (T), and audio-tactile (AT) estimates and predicted bimodal thresholds in the non-attentional **(C)** and attentional condition **(D)**. Error bars represent standard errors of the mean. In the non attentional condition **(C)** auditory thresholds are different from tactile and bimodal thresholds (^*^p < 0.05).

Figures 4A,B show average PSEs for the three bimodal conflicts plotted as a function of the audio-tactile conflict (Δ) in the non-attentional (Figure 4A) and attentional (Figure 4B) conditions. The two dashed lines describe the ideal performance in the case of auditory (light and dark red) or tactile (light and dark green) dominance. Black line and symbols represent observed PSEs data, gray line and symbols represent the model prediction. As predicted by the model, in the non-attentional condition bimodal PSEs follow the tactile conflict suggesting a tactile dominance. Average PSEs are equal to 0.45 ± 1.26 for the Δ = +7 conflict, 0.33 ± 0.79 for the Δ = 0 conflict and −2.05 ± 0.79 for the Δ = −7 conflict. Predicted PSEs were 2 ± 1.17 for the Δ = +7 conflict, 0.8 ± 1.08 for the Δ = 0 conflict and −0.5 ± 1.22 for the Δ = −7 conflict. We ran a Two-Way ANOVA to study differences between predicted and observed PSEs and between PSEs measured in different conflict conditions. In the non-attentional condition, we found a significant effect of the conflict [*F*_(2, 54)_ = 3.39; *P* = 0.04], but no significant differences between predicted and observed PSEs [*F*_(1, 54)_ = 2.11; *P* = 0.15] and no interaction between the two factors [*F*_(2, 54)_ = 0.20; *P* = 0.81]. In the attentional condition, we found no differences between PSEs across conflicts [*F*_(2, 54)_ = 0.11; *P* = 0.88], between predicted and observed PSEs [*F*_(1, 54)_ = 1.55; *P* = 0.21] and no interaction between the two factors [*F*_(2, 54)_ = 0.53; *P* = 0.58]. The effect of the conflict that we have found in the non-attentional condition confirms that participants founded their perceptual judgment mainly on one sensory modality. Additionally, the lack of differences between predicted and observed PSEs implies a good prediction from the MLE model. Figures 4C,D show the average thresholds for the audio (A), tactile (T) and audio-tactile (AT) estimates as well as the predicted bimodal thresholds. Since individual bimodal thresholds were similar across the three AT conflicts, for both the non-attentional [repeated measure ANOVA; *F*_(2, 27)_ = 1.03; *P* = 0.37] and the attentional condition [repeated measure ANOVA; *F*_(2, 27)_ = 2.37; *P* = 0.11], we calculated average bimodal thresholds for each participant.

For the non-attentional condition (Figure 4C), we compared unimodal and bimodal observed and predicted thresholds in a One-Way ANOVA and found significant difference [*F*_(3, 36)_ = 4.72; *P* = 0.007]. However, the Tukey HSD correction for multiple comparisons revealed a significant difference between auditory and bimodal thresholds (*P* = 0.04) but not between tactile and bimodal thresholds (*P* = 0.99) or between predicted and observed bimodal thresholds (*P* = 0.87). In the attentional condition (Figure 4D), the One-Way ANOVA reported a significant difference between unimodal and bimodal observed and predicted thresholds [*F*_(3, 36)_ = 3.22; *P* = 0.03]. The Tukey HSD correction showed no significant difference between tactile and bimodal thresholds (*P* = 0.07) between auditory and bimodal thresholds (*P* = 0.5) or between predicted and observed bimodal thresholds (*P* = 0.99). These results suggest that both optimal integration and sensory dominance are possible.

**Figure 5:**
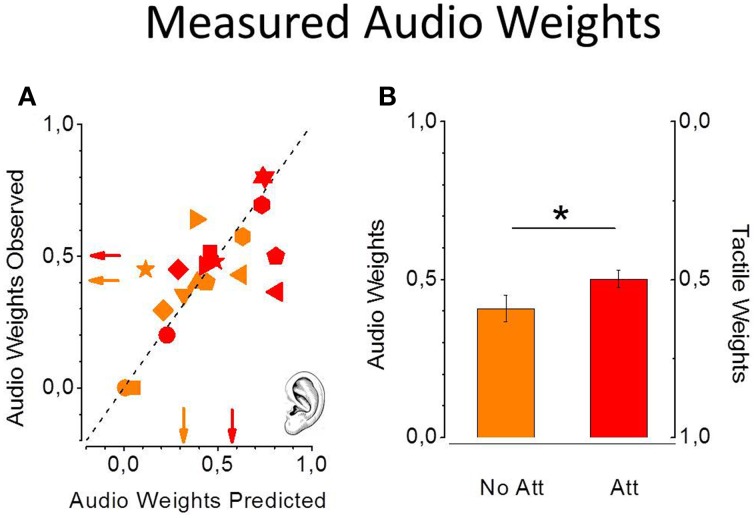
**Predicted and observed individual auditory weights from the 10 participants in the non-attentional (light red) and the attentional (dark red) condition (A)**. Standard errors were computed with bootstrap simulation. Data are all scattered on the equality line meaning perfect prediction from the MLE model. Measured average auditory weights are higher in the attentional condition **(B)**. (^*^p < 0.05).

Predicted and observed auditory weights are similar for all the participants in all the experimental conditions. Figure 5A shows observed individual audio weights plotted as a function of predicted individual audio weights in the non-attentional (light symbols) and attentional (dark symbols) conditions. All the data are scattered on the equality line suggesting that the model successfully predicted participants' performance. Moreover, observed and predicted audio weights are not statistically different [non-attentional condition: one tailed paired *t*-test, *t*_(9)_ = −0.75, *P* = 0.46; attentional condition: *t*_(9)_ = 0.81, *P* = 0.43]. More important, the average auditory weights are significantly higher in the attentional (dark red) condition with respect to the the non-attentional (light red) condition [one tailed paired *t*-test, *t*_(9)_ = −2.23, *P* = 0.02, see Figure 5B], as predicted by the model.

**Figure 6:**
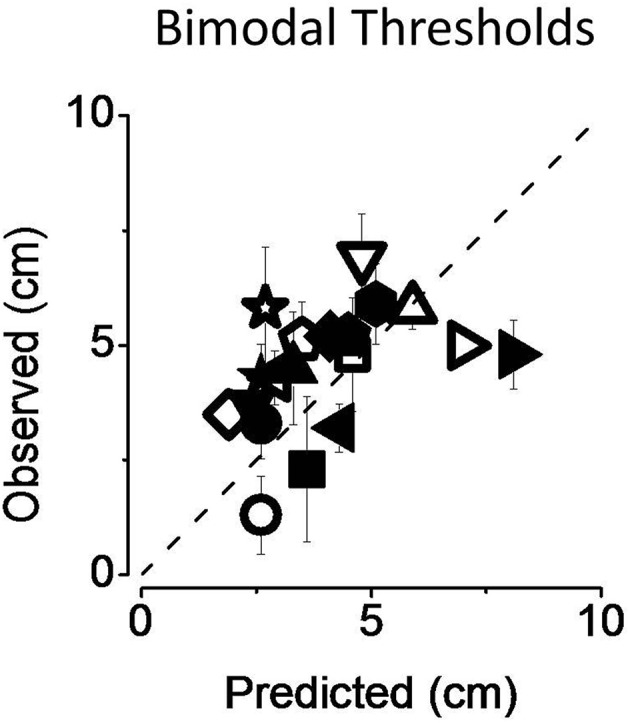
**Predicted and observed individual thresholds (A) for both the non-attentional (empty symbols) and the attentional condition (filled symbols)**. Standard errors were computed with bootstrap simulation. Data are well-predicted by the MLE model.

In Figure 6 we reported individual bimodal thresholds for both attentional (filled symbols) and non-attentional condition (empty symbols). Individual data are all scattered on the equality line and average thresholds are similar to those predicted by the MLE model.

## Discussion

In this study we investigated the effects of modality-specific attention on sensory reliability and on multisensory integration. We compared an attentional and non-attentional condition. In the attentional condition we found increased precision in the attended modality and a collateral change in cue weighting in the bimodal estimate. Our results support the idea that attention to a sensory modality can affect multisensory processing.

In the non-attentional condition participants performed the tactile task with higher precision than the auditory one. As predicted by the MLE model the tactile modality directed the final multisensory estimates. The dual auditory task (attentional condition) significantly improved auditory precision and increased unimodal weights. Also in this condition the bimodal estimates were successfully predicted by the MLE model, suggesting optimal integration. These results show that attention to sounds reduces auditory thresholds and that the improved auditory precision affects multisensory perceptual judgments and accuracy. However, in all the conditions we have tested, bimodal thresholds were not significantly different from either the best unimodal threshold or the MLE prediction, therefore, neither account could be rejected. Similarly, Alais and Burr (2004) and Gori et al. (2012) reported near-optimal integration with bimodal thresholds sometimes comparable to unisensory thresholds. The lack of significant improvement on precision may be due to several factors. As Alais and Burr (2004) have suggested, there may be an additional noise source at the level of bimodal combination not considered in the model or there may be correlations between the noise sources of the sensory modalities. The lack of statistical power might be another possible reason for failing to find strong support for MLE integration.

Our study appears to be in conflict with previous results from Helbig and Ernst (2008) that have recently examined the effect of a dual visual task on visuo-tactile integration. The distractor task used in Helbig and Ernst's experiment (2008) involved visual stimuli different from those used in the primary task. Participants had to evaluate the similarity between two sequences of letters presented just above the position of the visual stimulus of the primary size discrimination task. Authors reported that performing a dual visual task impaired precision in the visual modality but did not affect visual and tactile weights in visuo-tactile integration. Combining these two tasks might require extremely high cognitive resources. Indeed, authors found that also the tactile modality was slightly affected by the distractor task. Contrarily, in our task we asked participants to evaluate two different characteristics of the same stimulus: one spatial and the other temporal. Probably, the double-task that we used increases attention to the stimulus rather than withdrawing attention from it. Moreover, we presented the dual task randomly only in the 30% of the trials and analyzed the remaining 70% to be sure that participants were focused on the stimulus and not distracted by the secondary task.

Another possible explanation for the difference between our results and those from Helbig and Ernst (2008) is that spatio-temporal features of a stimulus may be encoded together in the brain. In both the studies participants were engaged in a double-task, a paradigm that generally increases the attentional load and results in a lower performance. Surprisingly, we found higher precision in the attentional condition than in the non-attentional one. Previous studies demonstrated that space and time are not processed separately but probably share similar neural mechanisms and similar cortical circuits (Burr and Morrone, 2006; Johnston et al., 2006). Under this perspective, performing a spatio-temporal dual task could not result in a reduction of spatial precision, but rather in an increased reliability of the attended stimulus.

Researchers have found that directing attention toward a particular region of space or to a sensory modality improves performance in several tasks. Yeshurun and Carrasco (1998) explored the effect of spatial attention on a texture segregation visual task and found attentional facilitation reflecting signal enhancement. Moreover, a neurophysiological study from Spitzer et al. (1988) reported that increasing the amount of effort required to perform a perceptual task, such as orientation or color discrimination, can affect information processing in the visual stream. When the task is more difficult the performance improves and neuronal responses to stimuli are larger and more selective. In a similar way, the attentional effort required by the dual task on the auditory stimulus used in our experiment might have improved the discriminative ability of the participants.

Our results are in line with several studies showing that attention to a sensory modality might affect perceptual estimates in multisensory tasks (Bertelson and Radeau, 1981; Warren et al., 1981; Alsius et al., 2005). For example Oruc et al. (2008) demonstrated that in crossmodal dynamic ventriloquism the motion signals from different sensory modalities are combined differently depending on modality-specific attention, but only when the susceptibility for capture between the two signals is comparable. Alsius et al. (2005) also showed that the McGurk illusion is severely reduced when participants are concurrently performing an unrelated visual or auditory task.

Yau et al. (2010) showed that auditory and tactile signals can be combined differently based on the perceptual task. Here we report a strong attentional modulation of AT integration. The current study adds an interesting contribution to the large body of empirical research supporting the idea that attention to modality can affect the process of multisensory integration. Moreover, although previous studies investigated AT integration with the MLE model in the temporal judgments (Ley et al., 2009) we explored optimal integration also in the spatial domain. Further studies might investigate whether this attentional effect can also reduce the visual “dominance” in an audio-visual or visuo-tactile spatial integration.

### Conflict of interest statement

The authors declare that the research was conducted in the absence of any commercial or financial relationships that could be construed as a potential conflict of interest.
